# Ruptured mature cystic teratoma with granulomatous inflammation masquerading as pseudomyxoma peritonei

**DOI:** 10.1007/s13691-025-00818-2

**Published:** 2025-10-23

**Authors:** Fenja Steinert, Jens Hölzen, Mazen Juratli, Jennifer Merten, Ralf Witteler, Eva Wardelmann, Andreas Pascher, Ann-Kathrin Eichelmann

**Affiliations:** 1 Department of General, Visceral and Transplant Surgery, Münster University Hospital, Münster, Germany; 2 Department of Gynecology and Obstetrics, Münster University Hospital, Münster, Germany; 3 Gerhard-Domagk-Institute of Pathology, Münster University Hospital, Münster, Germany

**Keywords:** Mature cystic teratoma, Ovarian neoplasm, Peritoneal carcinomatosis, Granulomatous inflammation, Cytoreductive surgery, Pseudomyxoma peritonei

## Abstract

Mature cystic teratomas are common benign ovarian neoplasms, but rupture is a rare complication, occurring in less than 5% of cases. Peritonitis with granulomatous lesions following rupture, mimicking peritoneal carcinomatosis, is extremely rare and is not typically reported as a complication. We report the case of a 48-year-old Caucasian female with lower abdominal pain and suspected four-quadrant peritonitis. A 7.9 cm teratoma was identified on CT scan. Exploratory laparoscopy revealed numerous adhesions and fibrinous exudate. Initially, cytoreductive surgery and hyperthermic intraperitoneal chemotherapy were recommended due to a highly suspected malignancy, initially thought to be pseudomyxoma peritonei. However, laparotomy revealed over 50 encapsulated mucoid micronodular lesions and pronounced adhesions throughout the entire abdominal cavity. Despite the macroscopic appearance, no evidence of malignancy was found in any of the numerous frozen sections sent for analysis. Histopathology confirmed a mature cystic teratoma with multifocal foreign body granulomas. No malignancy was detected. It is likely that multiple (silent) ruptures of the mature cystic teratoma were responsible for this unusually pronounced inflammatory response in the whole abdomen. This case report presents a rare and unusual complication of a mature cystic teratoma, where it mimics peritoneal carcinomatosis, leading to an initial misdiagnosis. While ruptured mature cystic teratomas are known, the granulomatous inflammation and peritonitis that mimicked pseudomyxoma peritonei have not been reported in the literature. This novel presentation could significantly affect diagnostic protocols in similar cases, as it provides insight into how granulomatous reactions can present similarly to malignancy, potentially leading to unnecessary aggressive treatments such as cytoreductive surgery and hyperthermic intraperitoneal chemotherapy.

## Introduction

Mature cystic teratomas (MCTs) are benign germ cell tumors with a low risk of malignant transformation, consisting of differentiated tissues derived from the three germ layers. Mature teratomas account for approximately 10–20% of ovarian neoplasms [1]. Although MCTs can occur at any age, they are most commonly found in women of reproductive age [2].

In 20% of cases, MCTs are asymptomatic. When symptoms do occur, they may include chronic abdominal pain or the presence of a palpable mass. Complications can present with a range of symptoms, and authors have emphasized the importance of a structured evaluation of these symptoms [1]. Common symptoms include acute abdominal pain, nausea, vomiting, fever, shock, and bleeding. The most frequent complication is ovarian torsion. Malignant transformation, though rare, can occur in 1–2% of cases, with the presence of necrosis and adhesions often serving as potential indicators of a malignant process [2].

Rupture of a teratoma is uncommon due to its thick cystic wall. However, rupture can occur under conditions such as increased intraperitoneal pressure from pregnancy, trauma, or torsion with necrosis of the cystic wall. MCT rupture is reported in 1.2–3.8% of cases and may result in either acute or chronic aseptic peritonitis [2].

## Case report

**Fig. 1:**
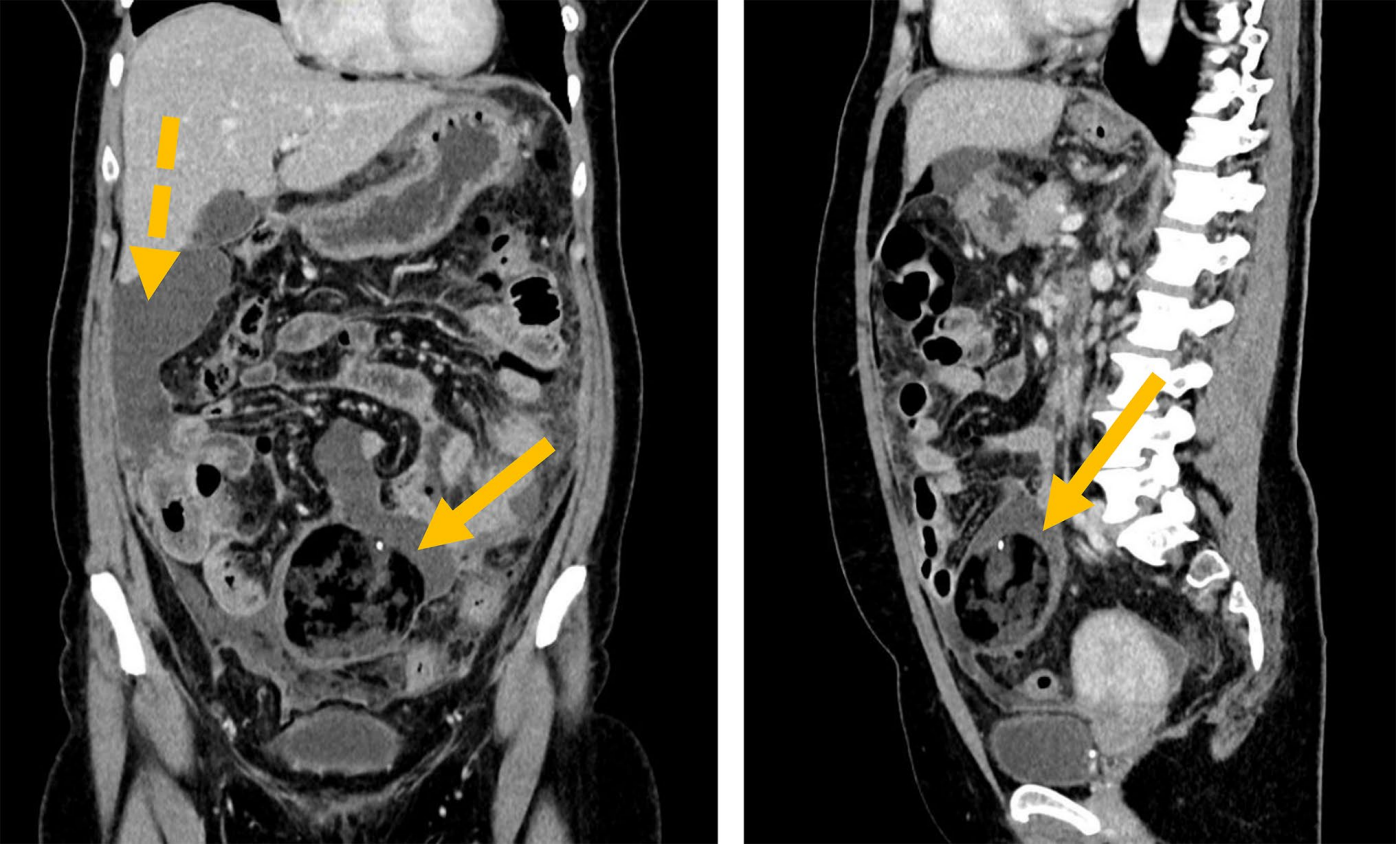
A computed tomography (CT) scan was performed at an early stage of the diagnostic process, following the ultrasound findings of perihepatic free fluids and an unclear lesion in the mid-abdomen. The solid arrow indicates the suspected teratoma, while the dashed arrow indicates the abscess-like formation

A 48-year-old Caucasian female patient was referred to our clinic with complaints of lower abdominal pain. A computed tomography (CT) scan revealed a strong suspicion of four-quadrant peritonitis and a 7.9 cm mass, likely a teratoma of the right ovary. In addition, the CT scan showed multiple abscess-like formations. The patient had initially presented to her general practitioner with acute, colicky lower abdominal pain (rated 5 on the numeric rating scale, NRS) that had persisted for 4 to 5 weeks. This pain was accompanied by occasional diarrhea and nausea. Administration of scopolamine butylbromide effectively relieved her pain. The patient had no known pre-existing conditions, no previous surgeries, consumed alcohol in moderation, was a non-smoker, and had not traveled abroad in the past 4 years. There was no family history of tumor disease. The patient was in good general and nutritional condition. During the abdominal examination, mild, diffuse tenderness was noted on palpation, without signs of abdominal rigidity. Laboratory tests showed elevated leukocyte and C-reactive protein (CRP) levels. The carbohydrate antigen (CA) 19-9 level was elevated at 100 U/mL (reference range: < 27 U/mL), while the CA 125 level was within the normal range at 30.7 U/mL (reference range: < 35 U/mL). Sonography revealed perihepatic free fluid and a suspected tumor in the mid-abdomen. A CT scan (Fig. 1) revealed the following findings:

- Urgent suspicion of four-quadrant peritonitis with abscess-like formations along the right hepatic border, paracolic groove, and pelvis.
- Wall thickening in the small and large intestines, as well as the appendix, most likely reactive in the context of peritonitis.
- Suspected teratoma of the right ovary, measuring 7.9 cm in diameter.
- No signs of ileus or hollow organ perforation.

Further diagnostic procedures included gastroscopy and colonoscopy, which did not reveal any significant underlying pathology. During a gynecological examination, a myoma was detected. Following this, the patient was referred to our consultation. Given the unclear nature of the mass in the lower abdomen, which could not be attributed to a specific organ, an exploratory laparoscopy was indicated. During the procedure, numerous interenteric adhesions were observed, as well as adhesions between the intestinal loops and the abdominal wall, severely limiting visibility. In addition, there was a pronounced fibrinous exudate in the abdominal cavity, and only partial detachment of the adhesions was possible. The tumor, located in the lower abdomen, measured approximately 10 to 12 cm in diameter, exhibited a firm, elastic consistency, and could not be definitively linked to any organ during the laparoscopy. Given the high risk of perforation, no histological sample was obtained. Based on the suspected diagnosis of pseudomyxoma peritonei, the interdisciplinary tumor board recommended cytoreductive surgery (CRS) followed by hyperthermic intraperitoneal chemotherapy (HIPEC).

**Fig. 2:**
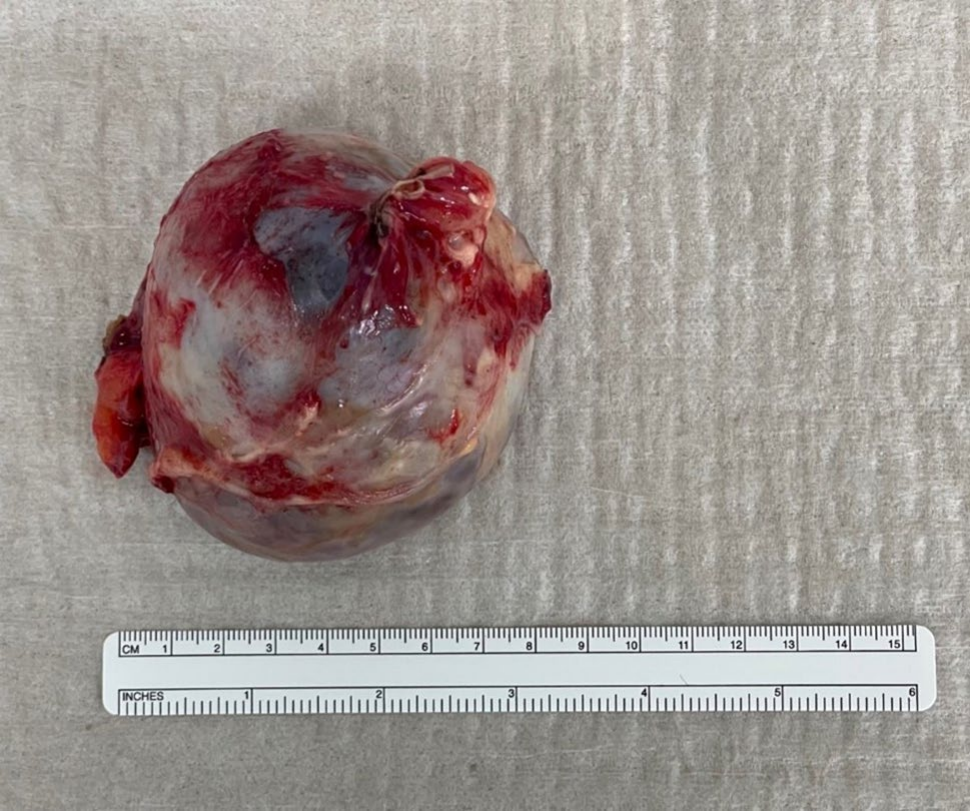
The tumor, measuring 8.1 cm in diameter, was located in the right ovary and classified as a mature teratoma

An exploratory laparotomy was subsequently performed. Intraoperatively, the tumor in the lower abdomen was found to originate from the right ovary. The tumor surface was intact. A right ovariectomy, along with resection of the tumor, was carried out (Fig. 2). A thorough examination of the abdominal cavity revealed numerous glassy micronodular lesions, with an estimated total of more than 50 lesions, some of which measured up to 3 × 3 cm. These lesions were found on the peritoneum of the diaphragm, the liver capsule, the omentum minus, abundantly on the omentum majus, the colon, the small intestinal mesentery, the small intestine, and within the small pelvis. These findings had resulted in the formation of multiple adhesions, which were already visible during the laparoscopic examination.

The primary challenge during the surgery was interpreting the macroscopic findings of the abdominal cavity. For all participating surgeons (both from visceral surgery and gynecology), the intraoperative appearance strongly suggested a malignant process. After an extensive period of adhesiolysis, the omentum majus was resected, and all macroscopically identifiable nodules were removed. A diagnostic appendectomy and left ovariectomy were also performed. Multiple samples of the intraabdominal fluid and the appendix were taken for frozen section analysis. None of these samples showed evidence of malignancy, which was inconsistent with the macroscopic appearance. Following an interdisciplinary discussion in the operating room, the surgeons decided not to proceed with CRS or HIPEC at that time, choosing instead to await the final histological results before deciding whether a two-stage CRS/HIPEC procedure would be necessary. The postoperative course was uncomplicated.

**Fig. 3:**
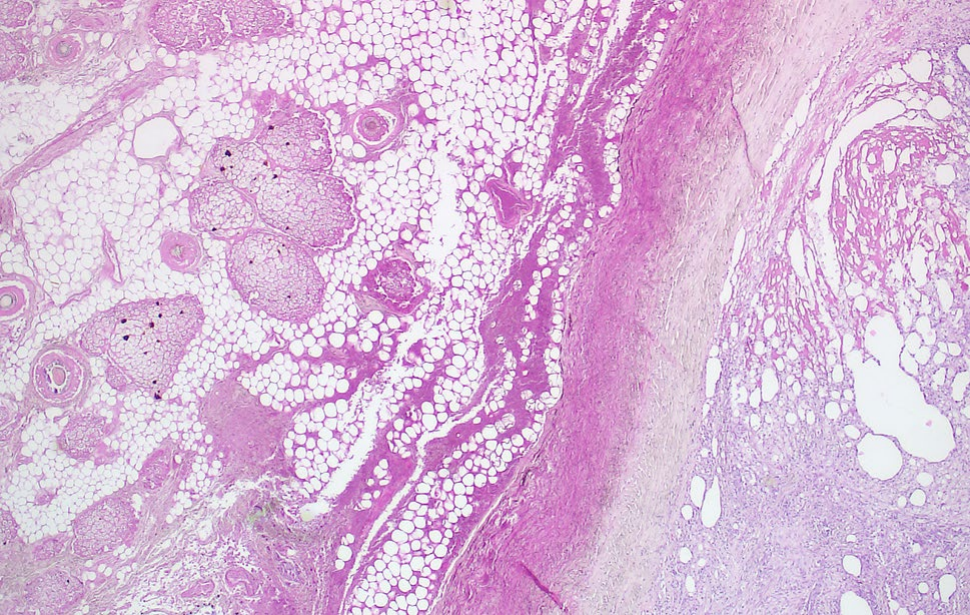
The histopathologic image of the mature cystic teratoma displays a portion of the cyst wall and an adjacent inflammatory reaction. The largely necrotic teratoma displayed skin-like structures with a multitude of hair follicles and sebaceous glands (original magnification × 5, H&E)

**Fig. 4:**
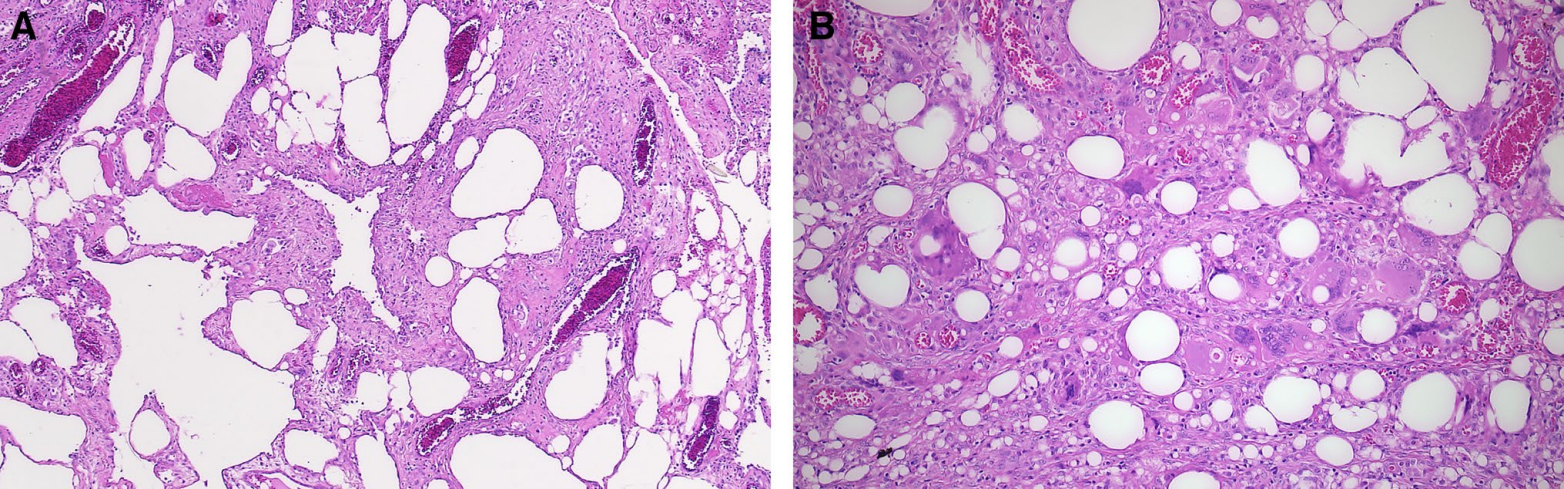
**A **and** B** Histopathological image of the lesions found throughout the entire peritoneal cavity. It shows a pronounced lipogranulomatous inflammatory reaction surrounded by multiple oil cysts (**A**) and multinucleated foreign giant cells (**B**). The diagnosis of a rupture of the teratoma as the cause of the inflammation is, therefore, very likely (original magnification ratio in A × 10, in B × 20; H&E)

The histopathological examination revealed the presence of an 8.1 cm cystic tumor attached to the right ovary. This tumor was diagnosed as a mature teratoma with extensive necrosis, which contained colon-like intestinal structures probably belonging to the attached appendix, sebaceous gland-rich skin-like tissue with numerous hair follicles (Fig. 3), and bony inclusions. Furthermore, a pronounced granulomatous giant cell-rich inflammatory reaction and partial mural necrosis of the cyst wall structures were observed. The mucoid glassy lesions demonstrated a pronounced histiocyte-rich granulomatous inflammation, accompanied by a considerable adipose tissue necrosis at all sampling localizations (Fig. 4). The morphological picture, with the unusual multinodular fibrinous, granulating, and xanthogranulomatous inflammation, correlated with the formation of foreign substance granulomas (Fig. 4). A silent rupture of the mature cystic teratoma of the right ovary, possibly also multiple times, appears to have been responsible for this unusually pronounced inflammatory reaction. The appendix did not demonstrate any evidence of malignancy. The typical picture of a pseudomyxoma peritonei was histologically not present. Understandably, the patient was not assigned to a two-stage CRS and HIPEC procedure.

**Fig. 5:**
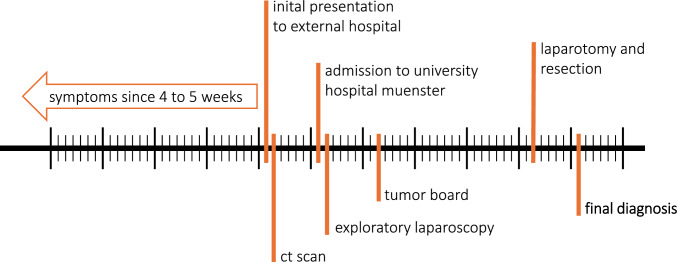
Chronological timeline of symptoms, diagnostic steps, and therapy until the final diagnosis was obtained

The diagnostic process, commencing with the initial presentation at the hospital and concluding with the definitive diagnosis of a benign process, spanned approximately 6 weeks (Fig. 5). This 6-week diagnostic process highlights the emotional burden placed on the patient during periods of uncertainty, particularly when malignancy is suspected. One year after the procedure, the patient remains symptom-free. After the complete resection of all cysts from the peritoneal cavity, it is reasonable to expect that the patient will not experience any further complications related to the disease or its treatment.

## Discussion

This case presents a rare and unusual complication of MCTs, where the clinical and intraoperative findings mimicked advanced-stage malignancy, specifically peritoneal carcinomatosis. The misdiagnosis was primarily due to the granulomatous inflammation and peritonitis that developed following a silent rupture of the MCT, a phenomenon that is exceptionally uncommon [1]. The granulomatous inflammation and peritonitis lead to a complex clinical presentation that closely resembled pseudomyxoma peritonei. The difficulty in distinguishing between benign and malignant processes highlights a critical challenge in clinical practice, even with the use of modern diagnostic imaging and histopathological techniques.

The treatment strategy for MCTs depends on several factors, including the patient’s age, family planning status, and the risk of malignant transformation. For postmenopausal women, the treatment of choice is typically resection with oophorectomy, which can be performed via laparoscopy or laparotomy. However, laparoscopy carries risks of intraoperative rupture, peritonitis, or the spread of malignant cells in the case of possible malignancy. The management of peritonitis due to a ruptured teratoma has been minimally addressed in the literature [2]. If rupture and subsequent peritonitis occur, surgical intervention is essential [3]. Unlike typical cases where a visible rupture of the teratoma results in immediate peritonitis, the silent rupture in this case caused a more subtle, yet pronounced, inflammatory response. It likely triggered the formation of granulomas throughout the peritoneum, contributing to the complex clinical presentation that mimicked malignancy. Only a few case reports have addressed this phenomenon (Table 1). The initial descriptions of granulomatous peritonitis were made by Van Orman and Mautner in 1951, and their findings were initially misinterpreted as tuberculous peritonitis [4]. More recently, Badru and colleagues reported that fewer than 80 cases have been documented since the original descriptions by Van Orman and Mautner [5]. In some cases, patients received aggressive surgical treatment [6] due to a suspected diagnosis of peritoneal carcinomatosis [7]. In many cases, granulomatous inflammation originating from MCT was mistaken for peritoneal carcinomatosis of ovarian origin, as both conditions share similar clinical and imaging features, such as an ovarian mass, the development of peritoneal metastases, and ascites [8–11]. In other cases, malignant transformation of a MCT was suspected [12]. However, in our case, the clinical presentation and intraoperative findings raised suspicion for pseudomyxoma peritonei. To the best of our knowledge, there has been no documented case in which the picture of granulomatous inflammation, following a teratomatous rupture, was mistakenly confused with pseudomyxoma peritonei.

**Table 1:** Summary of cases describing patients with mature cystic teratoma mimicking peritoneal carcinomatosis

Author	N	Age (years)	Sex	Surgery
Suprasert et al. (2004) [10]	2	50	Female	Laparotomy, hysterectomy, bilateral salpingo-oophorectomy, partial omentectomy
		53	Female	Laparotomy, bilateral salpingo-oophorectomy, partial omentectomy
Phupong et al. (2004) [11]	1	39	Female	Total hysterectomy, bilateral salpingo-oophorectomy
Minato et al. (2018) [12]	1	52	Female	No treatment
Badru et al. (2018) [5]	1	15	Female	Laparotomy, salpingo-oophorectomy, omentectomy
Tejani et al. (2020) [13]	1	32	Female	Bilateral salpingo-oophorectomy, omentectomy, pelvic lymph node dissections
Fatemi et al. (2021) [9]	1	23	Female	No treatment
Costachescu et al. (2024) [8]	1	61	Female	Laparotomy, left adnexectomy

Although the CT scan strongly suggested a teratoma, the diagnostic process still considered the possibility of malignancy, including malignant transformation, ovarian neoplasm with peritoneal carcinomatosis, or pseudomyxoma peritonei originating from the appendix or within the teratoma. Among these, pseudomyxoma peritonei appeared to be the most likely preoperative diagnosis due to CT findings, such as four-quadrant peritonitis with abscess-like formations and peritonitis. Unfortunately, the diagnostic laparoscopy failed to provide a definitive diagnosis. Despite thorough preoperative evaluation including tumor markers, imaging, and the mentioned diagnostic laparoscopy, a definitive diagnosis could not be established prior to laparotomy. Additional imaging with MRI would likely have provided only limited additional diagnostic value in this context.

Intraoperative findings, such as numerous micronodular glassy lesions and pronounced adhesions, initially suggested malignancy despite a non-suspicious frozen section. The elevated CA 19-9, the wall thickening of the appendix, and the presence of avital tissue in the histopathology were inconclusive. Morphologically, during laparotomy, there was no visible evidence of cyst wall rupture (histopathological analysis only revealed necrosis within the cyst wall). These factors contributed to the difficulty of intraoperative decision-making in this case.

This case highlights the importance of thorough evaluation and interdisciplinary discussions in ambiguous clinical situations. Despite advances in diagnostic techniques, distinguishing between benign and malignant tumors remains challenging, as demonstrated by the mature cystic teratoma with granulomatous inflammation and peritonitis that mimicked advanced malignancy. Clinicians should include granulomatous inflammation in the differential diagnosis of abdominal masses to avoid premature malignancy conclusions and prevent overtreatment. The decision to wait for the final histology report before proceeding with CRS/HIPEC was essential to avoid overtreatment of the patient. This case emphasizes the need for updated diagnostic protocols to avoid unnecessary aggressive treatments and stresses the value of careful pathological evaluation and interdisciplinary consultation. We recommend adding this rare complication to medical textbooks to guide future decisions and help prevent unnecessary cancer treatments.

## Data Availability

Data sharing is not applicable to this article as no datasets were generated or analyzed during the current study.
